# H_2_O_2_-responsive molecularly engineered polymer nanoparticles as ischemia/reperfusion-targeted nanotherapeutic agents

**DOI:** 10.1038/srep02233

**Published:** 2013-07-19

**Authors:** Dongwon Lee, Soochan Bae, Donghyun Hong, Hyungsuk Lim, Joo Heung Yoon, On Hwang, Seunggyu Park, Qingen Ke, Gilson Khang, Peter M. Kang

**Affiliations:** 1 WCU Department of BIN Fusion Technology, Chonbuk National University, Dukjin 664-14, Jeonju, Chonbuk 561-756, South Korea; 2 Polymer Fusion Research Center, Department of Polymer·Nano Science and Technology, Chonbuk National University, Dukjin 664-14, Jeonju, Chonbuk 561-756, South Korea; 3 Cardiovascular Institute, Beth Israel Deaconess Medical Center and Harvard Medical School, Boston, MA 02215, USA; 4 These authors contributed equally to this work.

## Abstract

The main culprit in the pathogenesis of ischemia/reperfusion (I/R) injury is the overproduction of reactive oxygen species (ROS). Hydrogen peroxide (H_2_O_2_), the most abundant form of ROS produced during I/R, causes inflammation, apoptosis and subsequent tissue damages. Here, we report H_2_O_2_-responsive antioxidant nanoparticles formulated from copolyoxalate containing vanillyl alcohol (VA) (PVAX) as a novel I/R-targeted nanotherapeutic agent. PVAX was designed to incorporate VA and H_2_O_2_-responsive peroxalate ester linkages covalently in its backbone. PVAX nanoparticles therefore degrade and release VA, which is able to reduce the generation of ROS, and exert anti-inflammatory and anti-apoptotic activity. In hind-limb I/R and liver I/R models in mice, PVAX nanoparticles specifically reacted with overproduced H_2_O_2_ and exerted highly potent anti-inflammatory and anti-apoptotic activities that reduced cellular damages. Therefore, PVAX nanoparticles have tremendous potential as nanotherapeutic agents for I/R injury and H_2_O_2_-associated diseases.

Ischemia/reperfusion (I/R) injury is cellular damage after reperfusion of previously ischemic tissues, and has been associated with several pathophysiological conditions including coronary arterial disease and stroke^1^,^2^,^3^,^4^,^5^. Reperfusion of blood flow to the ischemic tissues results in a large generation of toxic reactive oxygen species (ROS) and exacerbates initial tissue damages, which is the main culprit in the pathogenesis of I/R injury. In particular, hydrogen peroxide (H_2_O_2_) induces release of pro-inflammatory cytokines and triggers apoptosis, leading to the oxidative damage to tissues^6^,^7^. Therefore, targeting H_2_O_2_ as a diagnostic marker and therapeutic agent has tremendous potential.

Nanomaterials are being explored for many clinical applications in medicine^8^. Because nanomaterials possess greater permeability than other materials and could be formulated to respond to specific environmental factors, such as pH or temperature^9^,^10^, they hold great potential to be utilized in diagnosing and treating various medical conditions. In this study, we developed H_2_O_2_-responsive nanoparticles as I/R-targeted diagnostic and therapeutic agents and characterized their potential in animal models.

## Results

**Figure 1:**
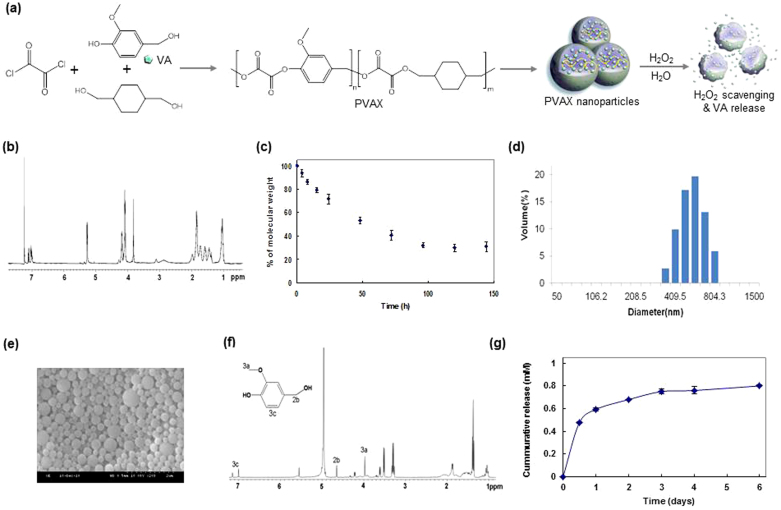
Chemical characterization of H_2_O_2_-activatable PVAX nanoparticles. (a) A schematic diagram of PVAX nanoparticle + synthesis and therapy for I/R. (b) ^1^H NMR spectrum of PVAX in CDCl_3_. ^1^H NMR in deuterated chloroform on a 400 MHz spectrometer: 7.0 ~ 7.3 (*m*, 3H, Ar), 5.3 (*m*, 2H OC**H**_2_-PhO-CH_3_), 4.1 ~ 4.2 (*m*, 4H, COOC**H**_2_CH), 3,8 (m, 3H, OC**H**_3_), 2.2 (*m*, 2H, C(C**H**_2_)_3_HO), 1.0 ~ 1.8 (*m*, 8H, Cyclic C**H**_2_). (c) Hydrolysis kinetics of PVAX under physiological conditions, N = 4/group. (d) A representative dynamic light scattering of PVAX nanoparticles suspended in PBS. (e) A representative SEM image of empty PVAX nanoparticles. (f) ^1^H NMR spectrum of PVAX after hydrolysis of 2 days in D_2_O. (g) Release kinetics of VA from PVAX.

We molecularly engineered PVAX to exploit the therapeutic activity of bioactive VA and the ability of peroxalate ester bonds to rapidly react with H_2_O_2_. VA is an active pharmaceutical ingredient in *Gastrodia elata* Blume, an herbal agent for brain ischemic injury and coronary heart diseases, and it exerts antioxidant, anti-inflammatory and anti-nociceptive activity^11^,^12^. PVAX was synthesized from a one-step polymerization of oxalyl chloride, VA and 1,4-cyclohexanedimethanol (Fig. 1a). PVAX possesses peroxalate ester bonds and VA covalently incorporated in its backbone. The chemical structure of PVAX was confirmed by ^1^H NMR and its molecular weight was determined to be ~12,000 *Da* with polydispersity of 1.6 (Fig. 1b). Despite its rapid hydrolysis kinetics with a half-life of ~36 h at pH 7.4 (Fig. 1c), PVAX was formulated into the solid nanoparticles under aqueous conditions because of its intrinsic hydrophobicity. PVAX nanoparticles were round spheres and their hydrodynamic diameter was determined to be ~500 nm (Fig. 1d and 1e).

PVAX was designed to release VA during its hydrolytic degradation under physiological conditions. In order to confirm the VA release from PVAX, PVAX was incubated in H_2_O at 37°C for 3 days and the supernatant was collected for ^1^H NMR. As shown in Fig. 1f, PVAX underwent hydrolytic degradation to release VA. We then investigated the release kinetics of VA from the PVAX nanoparticles under the physiological conditions. PVAX nanoparticles (1 mg/mL) released ~120 μg of VA during their hydrolytic degradation and more than half of the VA was released within 24 h (Fig. 1g). The rapid hydrolysis and VA release may provide considerable benefits for the treatment of diseases that require the fast onset of therapeutic action, such as acute liver injury and vascular diseases.

**Figure 2:**
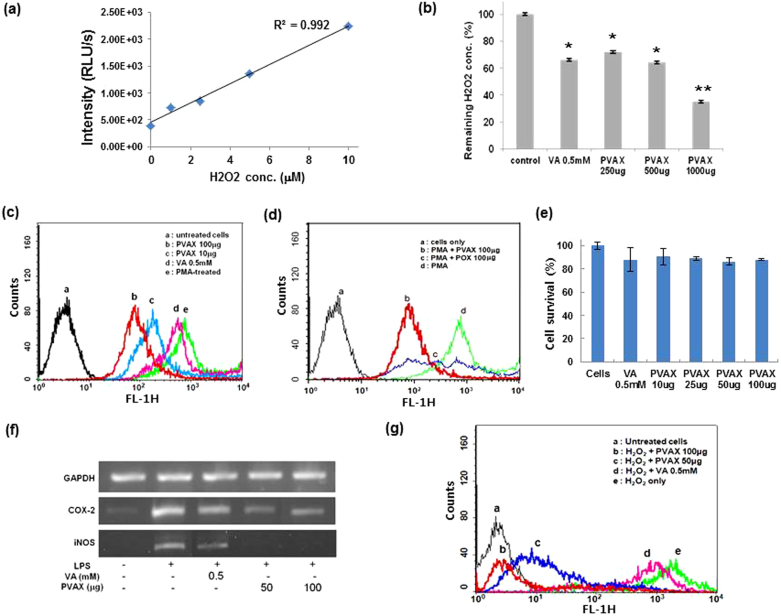
Anti-oxidant and anti-inflammatory properties of PVAX nanoparticles *in vitro*. (a) Sensitivity of chemiluminescent PVAX nanoparticles to H_2_O_2_. (b) Scavenging of H_2_O_2_ by PVAX nanoparticles. *, P < 0.01 versus Control; **, P < 0.01 versus VA, N = 4/group. (c) Inhibition of ROS generation by PVAX in PMA-stimulated macrophages. (d) Comparison in antioxidant activity of PVAX and POX. (e) Cytotoxicity of PVAX nanoparticles determined by MTT assay. VA = vanillyl alcohol. N = 4/group. (f) Anti-inflammatory activity of PVAX in LPS-stimulated macrophages. (g) Anti-apoptotic property of PVAX in H_2_O_2_-stimulated macrophages.

PVAX contains peroxalate ester bonds in its backbone, which are able to perform peroxalate chemiluminescence reaction in the presence of H_2_O_2_ and fluorophore. We therefore formulated chemiluminescent PVAX nanoparticles which encapsulate fluorophore rubrene (Rb) and investigated whether chemiluminescent PVAX nanoparticles could detect H_2_O_2_ by performing a three-component peroxalate chemiluminescence reaction. PVAX nanoparticles encapsulated with rubrene luminesced in the presence of H_2_O_2_, with a linear relationship between the chemiluminescence intensity and H_2_O_2_ concentration (Fig. 2a). PVAX nanoparticles should also scavenge H_2_O_2_ because peroxalate ester bonds in PVAX will react with H_2_O_2_ to generate dioxetanedione intermediates, which then should instantaneously decompose into CO_2_.

We also found that PVAX nanoparticles dramatically reduced the H_2_O_2_ concentration after 24 h in a dose-dependent manner, demonstrating that peroxalate ester bonds in PVAX undergo H_2_O_2_-mediated oxidation (Fig. 2b). Interestingly, VA also showed moderate H_2_O_2_ scavenging activity. Therefore, the highly potent H_2_O_2_ scavenging activity of PVAX nanoparticles is attributed to the combined effects of peroxalate ester bonds and VA released. The antioxidant activity of PVAX nanoparticles was investigated by measuring the level of intracellular ROS in RAW 264.7 macrophages stimulated with phorbol-12-myristate-13-acetate (PMA) using dichlorofluorescein-diacetate (DCFH-DA) as a marker of intracellular oxidative stress^13^,^14^,^15^. PMA treatment resulted in strong dichlorofluorescein (DCF) fluorescence in cells, which is indicative of oxidative stress in cells (Fig. 2c). VA (0.5 mM) slightly suppressed ROS generation, but PVAX nanoparticles remarkably inhibited the intracellular ROS generation. In order to further confirm the inhibitory effects of VA on ROS generation, we also prepared polyoxalate (POX) which has only aliphatic peroxalate ester bonds, but does not release VA. PVAX nanoparticles exhibited significantly more reduction in PMA-induced ROS generation than POX nanoparticles (Fig. 2d). These results demonstrate that the PVAX nanoparticles exert strong antioxidant activities by first scavenging intracellular H_2_O_2_ and then releasing VA that inhibits the further generation of ROS. In addition, cell toxicity study using MTT assay revealed that PVAX nanoparticles showed excellent biocompatibility (Fig. 2e).

We then evaluated the anti-inflammatory activities of PVAX nanoparticles in cells stimulated with lipopolysaccharide (LPS) by measuring the level of mRNA of genes related to inflammation. LPS stimulation induced remarkable expression of mRNA of pro-inflammatory mediators, such as inducible nitric oxide synthase (iNOS) and cyclooxygenase-2 (COX-2) (Fig. 2f). VA (0.5 mM) suppressed the expression of these pro-inflammatory mediators without changes in the level of glyceraldehyde 3-phosphate dehydrogenase (GAPDH), an internal control. However, PVAX nanoparticles exhibited the stronger inhibitory effect on the mRNA expression of iNOS and COX-2 in the LPS-stimulated cells than free VA.

We then investigated anti-apoptotic activities of PVAX nanoparticles in H_2_O_2_-stimulated cells (Fig. 2g). H_2_O_2_-stimulation activated the apoptotic cascade in cells, as evidenced in rightward shift in FITC-Annexin V fluorescence by flow cytometry, in good agreement with the literature^16^,^17^. In contrast, PVAX nanoparticles exerted inhibitory effects on H_2_O_2_-induced apoptosis in a dose-dependent manner. A dose of 100 μg of PVAX nanoparticles significantly (~71%) inhibited H_2_O_2_-induced apoptosis. Highly potent anti-inflammatory and anti-apoptotic activities of PVAX nanoparticles may be attributed to the combined effects of their antioxidant property and VA release. Taken together, PVAX nanoparticles demonstrate great synergistic therapeutic effects as a polymeric prodrug of VA as well as a highly potent H_2_O_2_-scavenging agent.

**Figure 3:**
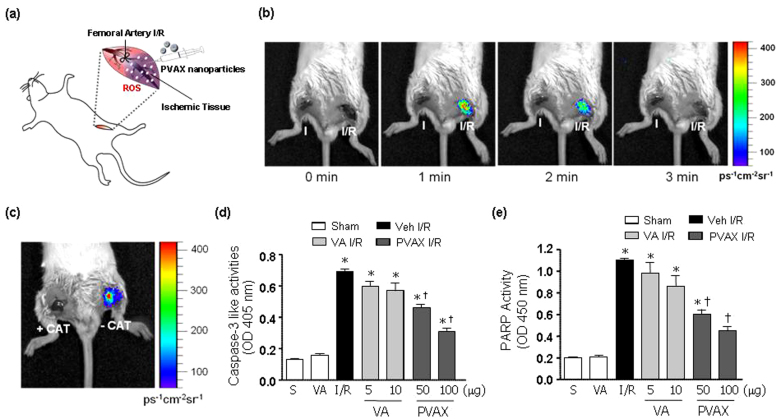
Bioimaging and apoptosis in hind-limb I/R model after PVAX *in vivo*. (a) A schematic diagram of hind-limb ischemia/reperfusion protocol. (b) *In vivo* imaging of PVAX/Rb after I/R in mouse hind-limbs. Reperfusion at different time points as indicated. Acquisition time = 30 sec/image. (c) *In vivo* imaging of PVAX/Rb with and without catalase. (d - e) Quantification of caspase-3 activities (d) and PARP-1 activity (e) after I/R with different concentrations of PVAX in gastrocnemius muscles. *, P < 0.05 vs Sham; †, P < 0.05 vs Veh + IR. N = 4–6/group.

To further extrapolate our *in vitro* findings, we investigated potential of H_2_O_2_-responsive PVAX nanoparticles as I/R-targeted therapeutic agents using a mouse model of hind-limb I/R injury (Fig. 3a). Initially, we tested H_2_O_2_-responsiveness of PVAX nanoparticles by determining whether chemiluminescent PVAX nanoparticles could image H_2_O_2_ generated endogenously during I/R. Ischemia was induced for 45 minutes in both limbs and chemiluminescent PVAX nanoparticles (PVAX/Rb) were directly injected just distal to the ligation sites (50 μg PVAX/Rb per site). Left hind limb was reperfused (I/R) but right hind-limb remained ligated (I). Chemiluminescent images were then captured at different time points. The site of I/R injury exhibited an intense chemiluminescence light emission lasting about 2 min after reperfusion demonstrating that chemiluminescent PVAX nanoparticles are capable of imaging endogenously generated H_2_O_2_ (Fig. 3b). Negligible chemiluminescent emission was observed at the site of ischemia only. To confirm whether PVAX nanoparticles are specific for H_2_O_2_, H_2_O_2_-degrading enzyme catalase was injected prior to the injection of PVAX nanoparticles. Pre-administration of catalase almost completely inhibited chemiluminescence emission at the site of I/R injury (Fig. 3c), further demonstrating that PVAX nanoparticles specifically detect H_2_O_2_. To our best understanding, this is the first study to report the imaging of endogenously generated H_2_O_2_ in I/R injury.

**Figure 4:**
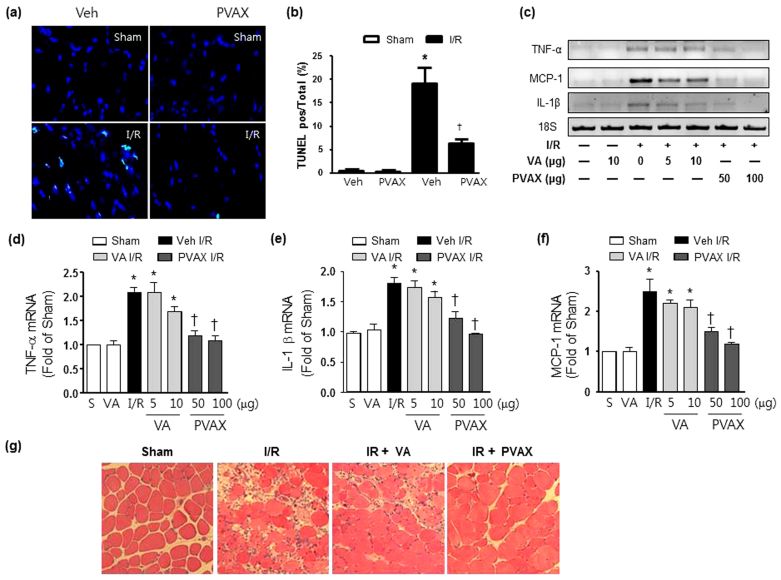
Cell death and inflammation in hind-limb I/R model after PVAX *in vivo*. (a) Representative TUNEL staining of gastrocnemius muscles after I/R with and without PVAX. (b) Quantification of apoptosis rate in gastrocnemus muscles after I/R with and without PVAX. *, P < 0.05 versus Sham; †, P < 0.05 versus Veh + I/R. N = 4–6/group. (c) mRNA levels of factors associated with inflammation after I/R with different concentrations of PVAX. (d–f) Quantification of TNF-α (d), IL-1β (e), and MCP-1 (f) mRNA levels after I/R with different concentrations of PVAX in HL I/R model. *, P < 0.05 vs Sham; †, P < 0.05 vs Veh + IR. N = 4-6/group. (g) Haematoxylin and eosin-stained histological sections of gastrocnemius muscle showed less damage and less apparent leucocyte infiltration in muscles in PVAX treated mice after I/R.

To test the therapeutic potential of PVAX nanoparticles for I/R injury, we injected PVAX nanoparticles in the gastrocnemius muscle after hind-limb I/R. Since I/R is known to induce apoptosis and cellular damage, we examined the ability of PVAX nanoparticles to inhibit the activation of polyADP ribose polymerase-1 (PARP-1) and caspase-3, both critical enzymes involved in apoptosis^18^,^19^,^20^. Since VA has been shown to have antioxidant and anti-inflammatory effect by itself, we injected the equivalent amount of VA (by weight, 1 VA = 10 PVAX) in a contralateral leg for comparison with PVAX. After I/R, there was significant activation of PARP-1 and caspase-3 (Fig. 3d and 3e). Treatment of PVAX nanoparticles showed significant inhibition of PARP-1 and caspase-3 activation by I/R in a dose dependent manner. In comparison, VA alone at the equivalent amounts contained in PVAX was able to modestly inhibit PARP-1 and caspase-3 activation only at the highest dose. Furthermore, treatment of PVAX nanoparticles demonstrated significant decrease in apoptotic myocytes after I/R compared to VA alone (Fig. 4a and 4b). There was also significant attenuation of various inflammation markers, such as tumor necrosis factor-alpha (TNF-α), monocyte chemotactic protein-1 (MCP-1), and interleukin -1β (IL-1β), and, after I/R in PVAX group compared to the I/R + vehicle group (Fig. 4c–4f). VA alone showed no significant suppression of these inflammatory markers. In addition, histological analysis showed that significant muscle damage induced by I/R injury was effectively blocked by PVAX nanoparticles (Fig. 4g). The higher therapeutic effects of PVAX than VA can be explained by the synergistic effects of H_2_O_2_-scavening peroxalate ester bonds and antioxidant and anti-inflammatory effect of VA. This study provides proof-of-concept that molecularly engineered PVAX nanoparticles are able to scavenge and image overproduced H_2_O_2_ and serve as I/R targeted therapeutic agents.

**Figure 5:**
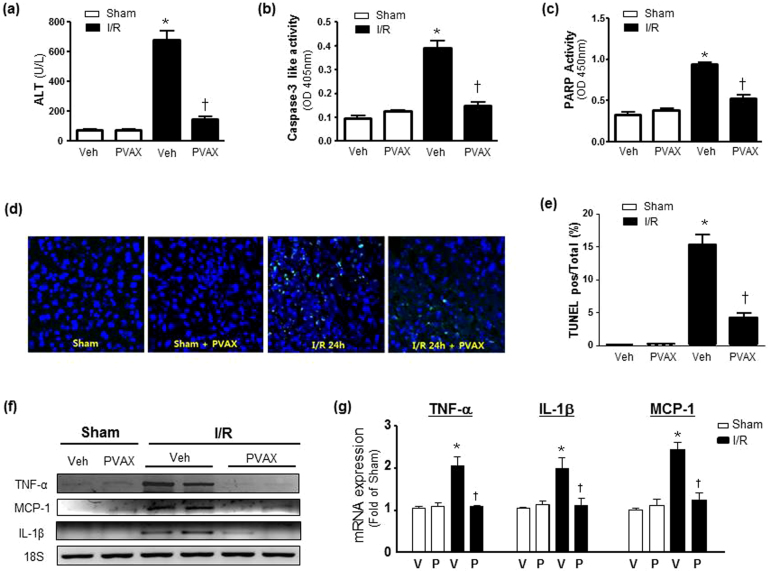
I/R-specific therapy using PVAX in hepatic I/R model *in vivo*. (a) Serum ALT level after 60 min ischemia and 6 h reperfusion with or without i.p. administration of PVAX. *, P < 0.05 versus Sham; †, P < 0.05 versus Veh + I/R. N = 4–6/group. (b–c) Quantification of caspase-3 activities (b) and PARP-1 activity (c) of the liver after I/R with and without PVAX. *, P < 0.05 versus Sham; †, P < 0.05 versus Veh + I/R. N = 4–6/group. (d) Representative TUNEL staining of liver after I/R with and without PVAX. (e) Quantification of apoptosis rate in liver after I/R with and without PVAX. *, P < 0.05 versus Sham; †, P < 0.05 versus Veh + I/R. N = 4–6/group. (f) Semi-quantitative PCR of mRNA levels of factors associated with inflammation after *i.p.* administration of PVAX after hepatic I/R. (g) Quantification of TNF-α, MCP-1 and IL-1β mRNA levels after I/R with or without PVAX in hepatic I/R model. *, P < 0.05 versus Sham; †, P < 0.05 versus Veh + I/R. N = 4–6/group.

In order to further demonstrate the therapeutic potential of PVAX nanoparticles in another clinically relevant setting, we used a mouse model of hepatic I/R. Hepatic I/R is a feature of many clinically important scenarios including liver transplantation^21^,^22^. In this model, PVAX (3 mg/kg) nanoparticles were injected intraperitoneally (*i.p.*) one hour prior to performing I/R in liver and again given just after reperfusion to evaluate their therapeutic potential. After 1 h of ischemia and 6 h of reperfusion, a dramatic increase in the serum alanine transaminase (ALT) activity, a marker of liver damage, was observed in a vehicle-treated group, compared with sham-operated controls (Fig. 5a). PVAX therapy significantly attenuated the serum ALT elevations induced by I/R. In addition, I/R markedly increased the PARP-1 and caspase activities in the liver, which was prevented by PVAX (Fig. 5b and 5c). Treatment with PVAX nanoparticles was also effective in decreasing apoptosis in hepatocytes after I/R compared to the saline I/R group (Fig. 5d and 5e). Furthermore, I/R significantly increased the mRNA expression of the proinflammatory cytokine TNF-α, MCP-1 and IL-1β (Fig. 5f and 5g). The I/R-induced acute hepatic proinflammatory responses, likely orchestrated by activated Kupffer and endothelial cells, were significantly attenuated by PVAX nanoparticles. Based on these findings, we conclude that PVAX nanoparticles have potential to be used as effective I/R-targeted nano-therapeutic agents and intrinsic antioxidant and anti-inflammatory properties of PVAX may further contribute to their overall beneficial effect during I/R injury.

**Figure 6:**
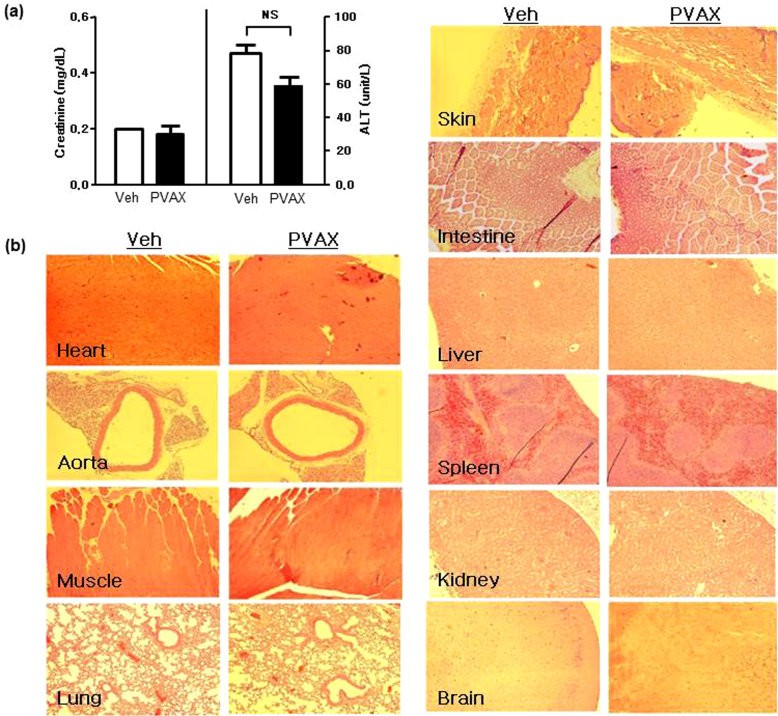
Safety profiles of PVAX. (a) Creatinine and ALT level after daily *i.p.* administration of PVAX (100 μg/day) for 7 days. NS = not significant. N = 4/group. (b) Representative Hematoxylin and Eosin (H&E)-stained tissue sections of various organs after PVAX. PVAX was administered *i.p.* at 100 mg/day for 7 days. N = 3/group.

Finally, to test the safety profile of PVAX, we administered 100 μg of PVAX nanoparticles daily for 7 days in mice. Serum tests for renal and hepatic function showed no significant abnormalities after 7 days (Fig. 6a). In addition, there was no obvious histological evidence of accumulated toxicity in the different organs associated with administration of PVAX nanoparticles for 7 days (Fig. 6b), demonstrating the excellent *in vivo* biocompatibility of PVAX.

## Discussion

Tissue damage is the most important determinant of morbidity and mortality after conditions associated with I/R injuries, such as myocardial infarction, vascular thromboembolic events, post cardiovascular surgery, transplant surgery, and post traumatic injuries^2^,^5^,^23^,^24^. Therefore, limiting cellular death is paramount for favorable outcomes in these conditions. Particularly, suppression of ROS overproduction during I/R using various antioxidants have been shown to effectively block the deleterious effects of ROS, such as apoptosis, in experimental settings *in vitro* and *in vivo*^4^,^25^. However, the beneficial effects of antioxidant therapy in human clinical studies have been disappointing due mainly to non- specific suppression of ROS in the body^26^,^27^. I/R-specific drug formulations would allow targeted release of drugs into specific areas or tissues that are undergoing a pathological process, leading to the enhanced therapeutic efficacy as well as decrease in related side effects. Therefore, H_2_O_2_-responsive PVAX nanoparticles may be able to serve as I/R targeted nanotherapeutic agents.

Ideal targeted drug delivery system would have combined target specificity with stimuli responsiveness to enhance the effects of the system^10^. Several such drug delivery systems have been generated that are responsive to pH, temperature, magnetic field, and concentrations of electrolytes or glucose^9^,^10^. PVAX nanoparticles are the first nanotherapeutic system that is shown in animal models to effectively treat tissues undergoing I/R injury by targeting endogenously generated H_2_O_2_ with high sensitivity and specificity.

In summary, we present novel I/R targeted nano-therapeutic agents based on molecularly engineered PVAX nanoparticles, which are sensitive and specific to H_2_O_2_. PVAX nanoparticles exhibit significant intrinsic antioxidant, anti-inflammatory, and anti-apoptotic activities both *in vitro* and *in vivo* models of I/R injury. We anticipate enormous potential of multifunctional PVAX nanoparticles for the H_2_O_2_-associated diseases, such as cardiovascular and neurovascular diseases.

## Methods

### PVAX synthesis

1,4-Cyclohexanedimethanol (21.96 mmol) and 4-vanillyl alcohol (5.49 mmol) were dissolved in 20 mL of dry tetrahydrofuran (THF), under nitrogen, to which triethylamine (60 mmol) was added dropwise at 4°C. Oxalyl chloride (27.45 mmol) in 25 mL of dry THF was added to the mixture dropwise at 4°C. The reaction was continued at room temperature for 6 h under nitrogen atmosphere and the resulting polymers were obtained through the extraction using dichloromethane and isolation by precipitating in cold hexane. The chemical structure of polymers was identified with a 400 MHz ^1^H NMR spectrometer and their molecular weight was determined using a gel permeation chromatography (GPC).

### Particle preparation and characterization

Fifty milligrams of PVAX dissolved in 500 μL of DCM was added to 5 mL of 10% poly-vinyl alcohol (PVA) solution. The mixture was sonicated using a sonicator (Fisher Scientific, Sonic Dismembrator 500) for 30 sec and a homogenizer (PRO Scientific, PRO 200) for 2 min to form a fine oil/water emulsion. The emulsion was added into 20 mL PVA 1% solution and further homogenized for 1 min. The remaining solvent was removed using a rotary evaporator. PVAX nanoparticles were obtained by centrifuging at 11,000 g for 5 min at 4°C, washing with deionized water twice and lyophilizing the recovered pellet. The morphology and size of PVAX nanoparticles were observed by a scanning electron microscopy (SEM, S-3000N, Hitachi, Japan) with accelerating voltage of 10 Kv. The hydrodynamic size of PVAX nanoparticles was determined using a particle analyzer (ELS-8000, Photal Otsuka Electronics, Japan).

### Release kinetics of vanillyl alcohol from PVAX nanoparticles

PVAX nanoparticles (5 mg) were added into 5 mL of phosphate buffer solution (pH 7.4) and the particles suspension was incubated at 37°C with mechanical stirring. At appropriate intervals, the solution was centrifuged at 2000×*g* for 20 sec and the 1 mL aliquot of supernatant was taken and replaced with an equal volume of fresh phosphate buffer solution. The concentration of vanillyl alcohol in the supernatant was measured using a UV spectrometer (S-3100, Scinco, Korea) and the release kinetics was determined by comparing the concentrations of vanillyl alcohol standard solutions.

### Cytotoxicity assay and detection of H_2_O_2_

3-(4,5-dimethylthiazol-2-yl)-2,5-diphenyltetrazolium bromide (MTT) assay was performed to evaluate the cytotoxicity of PVAX nanoparticles. Mouse macrophage RAW 264.7 cells were cultured at a density of 1 × 10^6^ cells/well in a 24 well plate containing 1 mL of culture medium for 24 h. Cells were treated with various amounts of nanoparticles and incubated for 24 h. Each well was given 20 μL of MTT solution and were incubated for 4 h. Two hundred microlitters of dimethyl sulfoxide (DMSO) was added to each well to dissolve the resulting formazan crystals. After 30 min of incubation, the absorbance at 570 nm was measured using a microplate reader (E-Max, Molecular Device Co. US). The cell viability was obtained by comparing the absorbance of nanoparticles-treated cells to that of control cells.

### Flow cytometry

RAW 264.7 cells (1 × 10^6^ cells) were cultured for 24 h. Cells were treated with vanillyl alcohol and PVAX nanoparticles for 4 h. Phorbol-12-myristate-13-acetate (PMA, 0.5 mg) was given to cells for the generation of reactive oxygen species and 20 h later cells were treated with DCFH-DA. Cells were also treated with 100 μM of H_2_O_2_ for 4 h to induce apoptosis and treated with Annexin-V labeled with FITC for 15 min. Flow cytometry was performed with a Flow Cytometry Caliber (Becton Dickinson, US).

### Reverse transcription-polymerase chain reaction (RT-PCR)

The RAW 264.7 cells were plated at a density of 2 × 10^5^ in 24 well tissue culture plates. The cells were pretreated with 0.5 mM vanillyl alcohol and a various amount of PVAX nanoparticles for 24 h and then treated with 1 *μ*L of LPS (1 mg/mL) for 12 h. Total cellular RNA was isolated using 1 mL of TRIzol (Invitrogen, Life Technologies Co, Groningen, Netherlands) according to the manufacturer's instructions. One microgram of total RNA was reverse-transcribed into cDNA using oligo (dT) primer (Invitrogen), 5X First Strand buffer (Invitrogen), dNTP (Gibco), RNase inhibitor (Invitrogen), SuperScript II (Invitrogen), and RNase H reverse transcriptase (Invitrogen). PCR was performed on aliquots of the cDNA preparations to detect iNOS, COX-2, IL-1β and GAPDH (the internal standard) gene expressions. The PCR primers used in this study are listed below: sense iNOS, 5′-AAT GGC AAC ATC AGG TCG GCC ATC ACT-3′, anti-sense iNOS, 5′-GCT GTG TGT CAC AGA AGT CTC GAA CTC-3′; sense COX-2, 5′-GGA GAG ACT ATC AAG ATA GT-3′, anti-sense COX-2, 5′-ATG GTC AGT-AGA CTT TTA CA-3′; sense GAPDH, 5′-TGA ACG GGA AGC TCA CTG G-3′, anti-sense GAPDH, 5′-TCC ACC ACC CTG TTG CTG TA-3′. The PCR primers used for animal tissues are listed below: sense TNF-α, 5′-CCT CAG CCT CTT CTC CTT CCT-3′, anti-sense TNF-α, 5′-GGT GTG GGT GAG GAG CA-3; sense IL-1 β, 5′-CTG AAA GCT CTC CAC CTC-3′, anti-sense IL-1 β reverse, 5′-TGC TGA TGT ACC AGT TGG GG-3′; sense MCP-1 forward, 5′-CCC CAC TCA CCT GCT GCT ACT-3′, anti-sense MCP-1 reverse, 5′-GGC ATC ACA GTC CGA GTC ACA -3′; sense 18S forward, 5′-GTT ATG GTT CCT TTG TCG CTC GCT C-3′, anti-sense 18S reverse, 5′- TCG GCC CGA GGT TAT CTA GAG TCA C-3′. After amplification, portions of the PCR reactions were electrophoresed on 2% agarose gel, and visualized under UV after ethidium bromide staining.

### Animal surgeries

Hind limb I/R surgeries were performed in 15–16 week old male mice (Charles River Laboratory, Wilmington, MA). After mice were anaesthetized, femoral artery was identified and tied around a specialized 30G-catheter with a 7-0 silk suture. The animal remained under anesthesia for a specified duration of ischemia. Reperfusion was achieved by cutting the suture and re-establishing arterial blood flow. Sham operated mice underwent the same procedure without femoral artery occlusion/reperfusion. Mice were sacrificed and analyzed at 2 days for biochemical/molecular studies, and at 2 weeks for histological analysis.

Hepatic I/R surgeries were done in 10–12 week-old male mice (Charles River Laboratory, Wilmington, MA). One hour prior to anesthesia, PVAX group mice received 50 μl of PVAX nanoparticle through intraperitoneal route. Mice in saline group received same volume of normal saline. After one hour, all mice were anaesthetized with intraperitoneal injection of mixed solution of Ketamine and Xylazine (8:1 ratio). Midline incision was performed for laparotomy. After identifying the portal triad and biliary tree, the main trunk of hepatic artery and portal vein were clamped with vascular clip except for the vasculatures to the right lower lobe to achieve ischemic injury to approximately 70% of the liver. 60 minutes of ischemic time was allowed in I/R group mice. No vascular clamp was done for Sham group mice. After one hour, reperfusion was achieved by releasing the vascular clip. At that moment, additional 50 μl of PVAX nanoparticle was given to PVAX group, and same amount of saline was administered for Saline group. Then the midline incision was closed with 5-0 black silk suture. Half of the mice in each group were sacrificed at 6 hours for inflammatory activities (TNF-α, IL-1β, and MCP-1) and serum ALT measurements, and the rest were sacrificed at 24 hours for apoptotic activities (caspase-3, PARP-1). All experimental procedures were approved by the Institutional Animal Care and Use Committee of Beth Israel Deaconess Medical Center.

### Chemiluminescence imaging

In vivo bioluminescence imaging was carried out with a Xenogen IVIS 200 imaging system (Caliper LS, Hopkinton, MA). Images and measurements of luminescent signals were acquired and analyzed using Living Image software. Balb/c mice (Orient Bio, Korea) were anesthetized using 1–3% isoflurane, and placed onto the warmed stage inside the camera box. The animals received continuous exposure to 1–2% isoflurane to sustain sedation during imaging. All experiment procedures were performed with the approval of Chonbuk National University Animal Care Committee.

### Statistical analyses

Calculations and statistics were performed using GraphPad 5.0 software (GraphPad Software Inc., La Jolla, CA). Statistical analysis was carried out using the one-way analysis of variance (ANOVA) and Bonferroni's tests for post hoc differences between group means. Statistical significance was defined as P < 0.05. Results are presented as mean ± the standard error of the mean (SEM).

## Author Contributions

D.L., S.B. and P.M.K. designed and performed experiments, analyzed data and wrote the paper; D.H., H.L., J.H.Y., O.H., S.P., Q.K. performed experiments and analyzed data; G.K. supervised experiments and analyzed data.
